# Supplementary material for: Autonomy-supportive sexual health communication and sexual health behaviors for black and Latino MSM in the House Ball Community: Protocol for a social network analysis and exploratory structural equation model
Source: PLoS One. 2023 Feb 3;18(2):e0276350. doi: 10.1371/journal.pone.0276350 (PMC9897560; doi:10.1371/journal.pone.0276350)
Supplement: S1 Data — (DOCX) [file pone.0276350.s001.docx]

**S1 Data Validation Protocol**

**Ensuring Quality and Validity of Survey Responses**

The following items will be reviewed for each survey submission to ensure validity:

1. Proper response to attention check screener items;
   - Total of 4 items embedded in two question grids throughout the survey
2. Time to take survey; should be at least 10 minutes
3. Review of email submitted for compensation
4. Referral Identification validation for referred participants
   - Participants who are referred to the survey by seed participants will receive an email containing a customized link. The link will be embedded with a unique referral identification code linking their survey response to the seed participant by whom they were referred. These referral identification codes are captured in REDCap and invisible to participants to maintain anonymity. Compensation will be issued to referred participants only if the unique referral code matches that of the seed participant who referred them into the study.
5. Internet Protocol (IP) address review
   - Duplicate or multiple IP addresses
     - IP addresses from recruitment spaces will be captured with a ‘sample’ survey taken by the co-principal investigator prior to targeted recruitment. This IP address will be documented to prevent unjust removal of responses.
   - IP address location is outside of United States

The chart below will be used to document and take appropriate action based on the number and severity of validity flags for each completed survey. If a survey is assigned 6 or more points, it will be flagged for removal and a subsequent email will be sent notifying the participant.

| **Validity Item** | **Validation Flag** | **Action** |
| --- | --- | --- |
| 1. Proper response to attention check screener items | - Incorrect response to 1 or more items | 1 point issued for each incorrect response (up to 4 points total) |
| 1. Survey response time | - Less than 10 minutes | 1 point issued |
| 1. Compensation email address | - Repeat emails - Same email with different domains | - First valid response accepted - Remaining responses discarded; email notification sent to email listed in compensation field of response set. |
| 1. Referral ID validation | - Referral code unavailable or untraceable to a seed participant | - 1 point issued for unavailable or untraceable referral code |
| 1. IP address review | - IP address located outside of the United States | Participant and associated data removed from database; participant notified via email |
